# Metal artifact reduction in kV CT images throughout two-step sequential deep convolutional neural networks by combining multi-modal imaging (MARTIAN)

**DOI:** 10.1038/s41598-022-25366-0

**Published:** 2022-12-02

**Authors:** Hojin Kim, Sang Kyun Yoo, Dong Wook Kim, Ho Lee, Chae-Seon Hong, Min Cheol Han, Jin Sung Kim

**Affiliations:** Department of Radiation Oncology, Yonsei Cancer Center, Heavy Ion Research Institute, Yonsei University College of Medicine, 50-1 Yonsei-Ro, Seodaemun-Gu, Seoul, 03722 Korea

**Keywords:** Medical imaging, Biomedical engineering

## Abstract

This work attempted to construct a new metal artifact reduction (MAR) framework in kilo-voltage (kV) computed tomography (CT) images by combining (1) deep learning and (2) multi-modal imaging, defined as MARTIAN (Metal Artifact Reduction throughout Two-step sequentIAl deep convolutional neural Networks). Most CNNs under supervised learning require artifact-free images to artifact-contaminated images for artifact correction. Mega-voltage (MV) CT is insensitive to metal artifacts, unlike kV CT due to different physical characteristics, which can facilitate the generation of artifact-free synthetic kV CT images throughout the first network (Network 1). The pairs of true kV CT and artifact-free kV CT images after post-processing constructed a subsequent network (Network 2) to conduct the actual MAR process. The proposed framework was implemented by GAN from 90 scans for head-and-neck and brain radiotherapy and validated with 10 independent cases against commercial MAR software. The artifact-free kV CT images following Network 1 and post-processing led to structural similarity (SSIM) of 0.997, and mean-absolute-error (MAE) of 10.2 HU, relative to true kV CT. Network 2 in charge of actual MAR successfully suppressed metal artifacts, relative to commercial MAR, while retaining the detailed imaging information, yielding the SSIM of 0.995 against 0.997 from the commercial MAR.

## Introduction

Computed tomography (CT) is a viable imaging modality for radiation therapy, which is used to contour tumors and normal organs, perform treatment planning with dose calculation, and conduct image-guidance for the actual treatment. The tube voltage in generating X-ray ranges from 80 to 150 kV for planning fan-beam CT and cone-beam CT (CBCT) in general. It benefits from higher contrast due to the photoelectric effect prevalent in the kV energy range. However, the kV-CT should be substantially sensitive to materials with a high atomic number, such as metal^1–4^. Many of the patients have metal implanted for bilateral hip prosthesis, fixation of the spine, dental filling, etc. The high atomic materials yield severe streak artifacts, beam hardening, and photon starvation around those in the reconstructed CT images. The presence of metal artifacts mainly impairs both accuracy and efficiency of treatment planning as the metal-contaminated image could preclude accurate contouring and dose calculation in treatment planning, leading to the necessity of labor-intensive density override.

In an effort to minimize the imaging artifacts, a variety of metal artifact reduction (MAR) algorithms have been developed^5–25^. The most widely used technique is to identify and remove the metal-associated region in projection (processed projection), finally replacing the missing region by linear interpolation, also called the in-painting method^9–12^. However, the linear interpolation in projection datais likely to neglect the edge information in metal trace, and introduce a new imaging artifact due to lack of smooth transition between the original and missing regions. To mitigate the transition effect throughout the linear interpolation, normalized MAR (NMAR) by employing prior image information was proposed^13–17^. It was hypothesized that normalizing the processed projection after removing the metal-associated region would be able to attain less modulated intensity distribution, which benefit for suppressing a possibility of introducing new imaging artifacts. It could reduce the drawback of the conventional linear interpolation, while the thresholding-based technique might still lead to wrong tissue classification in generating the prior image necessary for the relevant normalization. The iterative reconstruction applied to the processed projection was an alternative to it, which attempts to control the potential imaging artifacts by regularizing such edge-preserving transformations as total-variation, and wavelet with the statistically weighted projection information^18–27^. Due to highly complicated pattern of metal-driven artifacts in clinical cases, a single technique could not completely address the pitfalls thus far, so no standard technique has been built yet.

Deep learning is a recent breakthrough that shows promising results in many diagnostic and therapeutic applications^28,29^. Convolutional neural networks (CNN) are powerful deep learning techniques inimage processing^30–35^. Several studies have developed deep learning-based algorithms for MAR in the projection or image domain that focused on improving the accuracy of linear interpolation and normalization methods^36–41^. Applying the deep neural network to artifact correction including MAR has been considered challenging, as most of the currently available deep neural networks are subject to supervised learning that requires both metal artifact containing image (input) and metal artifact-free image (output) for the network training. A recent study suggested generating metal artifact-free digital phantom images from genuine kV CT images by manipulating the projection data of the metal-free CT images^39^. However, the digital phantom image could not completely simulate the actual metal artifacts occurring in the conventional kV CT image.

This work presents a novel framework for MAR in kV CT image based on two-serial convolutional neural networks, named Metal Artifact Reduction throughout Two-step sequentIAl Networks (MARTIAN). To address the underlying challenge from the supervised learning system above, the principle of multi-modal imaging was used to generate artifact-free synthetic kV CT images from a different imaging modality (other than kV CT) that is robust to the metal artifact. To achieve this, mega-voltage (MV) CT was employed to generate artifact-free kV CT in the first deep neural network. The subsequent network used the resulting artifact-free image for actual artifact correction.

## Methods

### Utilizing multi-modal images to overcome challenges

**Figure 1:**
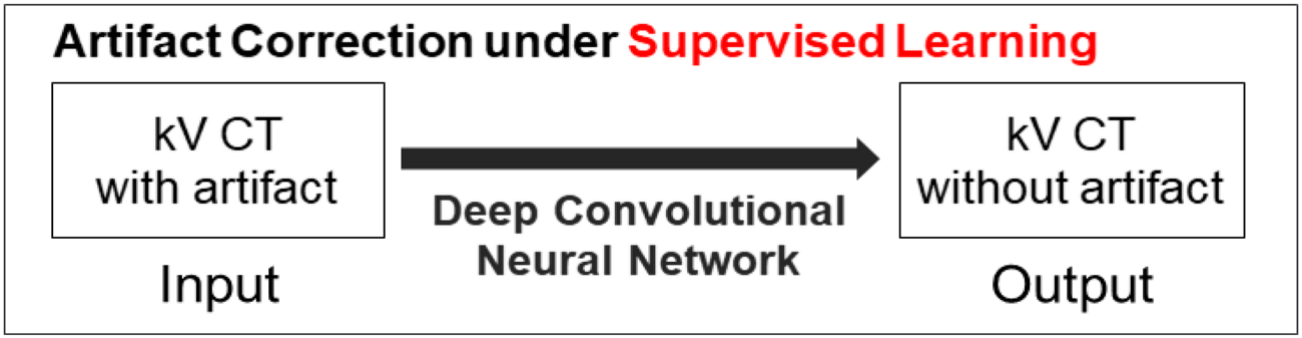
Difficulty of artifact correction by deep learning under supervised learning: artifact-free kV CT matching against artifact-containing kV CT image is not provided.

As illustrated in Fig. 1, the current deep convolutional neural networks, mostly based on supervised learning, require both metal artifact-contaminated and artifact-free images for metal artifact correction. That is a challenging issue in collecting such clinical kV CT images for network training. Employing multi-modal imaging could overcome the challenge, referring to the fact that each medical imaging modality has distinct physical characteristics. It implies that one imaging modality is susceptible to a particular artifact, while the other is robust to it. When applying it to metal artifacts, the mega-voltage CT (MV CT) should be far more insensitive to the artifact than the kV CT images, as the photoelectric effect diminishes with the energy increase. Thus, it is expected that the MV CT has the potential to be used to produce metal artifact-free images. In radiotherapy, various imaging modalities are being acquired to enhance treatment accuracy. Though it has been replaced with kV CBCT recently, some radiotherapy units e.g. TomoTherapy scan MV CT for image-guiding purposes by matching it against the planning kV CT images. The notion of multi-modal imaging was brought into the new deep neural network-based framework for MAR.

### Two-stage deep neural network for MAR

**Figure 2:**
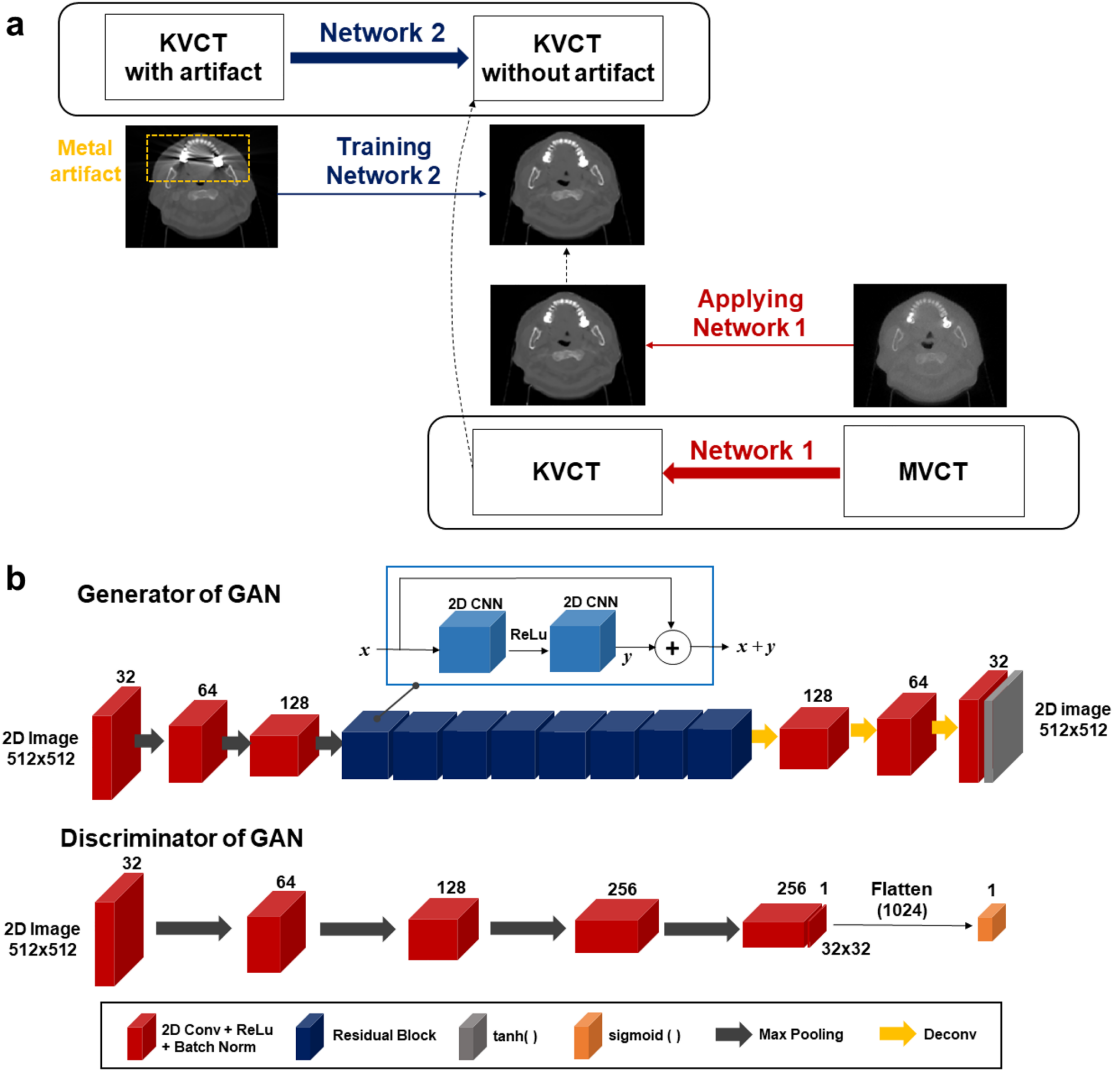
(**a**) Proposed MAR in kV CT images throughout Two-step sequential Network (MARTIAN), and (**b**) Network architectures of generator (with no skip connection) and discriminator used for GAN.

Image translation is one of the most frequent applications of the CNNs, which can be also applied to generating the synthetic kV CT images from the MV CT images. As the MV CT is a metal-artifact-free imaging modality, the resulting translated images are likely to exclude metal artifacts. The generated artifact-free images could be the output images of the deep neural network against the metal-contaminated input images. This is the basic motivation of the proposed framework, consisting of the two-step sequential deep convolutional neural networks for MAR (MARTIAN), as illustrated in Fig. 2a The first network, defined as Network 1, was designed to translate MV CT to kV CT images, generating synthetic metal-artifact-free kV CT images from MV CT images. The resulting artifact-free kV CT images were transmitted to the output of the subsequent network, denoted by Network 2. By setting the metal-contaminated kV CT images as input against the corresponding metal-artifact-free images, Network 2 plays a role in reducing the metal artifact in the end. Namely, Network 2 required the artifact-free kV CT images, produced from Network 1, corresponding to the metal contaminated images Once generated, Network 1 would be no longer engaged in MAR process. Network 2 is the only network employed to conduct the artifact-reducing task.

Two sequential networks technically conducted image translation. As the generative adversarial network (GAN)^45^ was demonstrated to work out for image translation, the GAN architecture was applied to both networks. Figure 2b shows the network architectures of the discriminator with convolution blocks only and a generator consisting of convolution blocks and residual blocks^46^. The generator had three layered down- and up-sampling blocks and 8-layered residual blocks at the bottleneck, while the discriminator had five layered down-sampling blocks, followed by a sigmoid function. Indeed, U-Net and its variations are currently the most popular. The network with skip-connection, generally used for U-Net, was not engaged in the generator as it did not fully suppress the streak artifact in the generated kV CT images. The CNN without skip-connection embedded in GAN architecture was more powerful in this application. All the learning tasks were performed with a 2D paired image dataset. The loss function of the generator was defined as a combination of adversarial loss of the conventional GAN, L1-loss, and structural similarity (SSIM) loss, as expressed in Eq. (1).

1 $$\begin{aligned} L_{GAN} & = \left[ {Adversarial\; Loss \, of \, GAN} \right] \\ & { + }10 \times \left[ {\frac{1}{N}\sum\limits_{i = 1}^{N} {\left( {|G(M_{i} ) - K_{i} |)} \right)} + \left( {1 - + SSIM(G(M),K)} \right)} \right] \\ & \quad {\text {where}}\quad SSIM(x,y) = \frac{{(2\mu_{x} \mu_{y} + c_{1} )(2\delta_{xy} + c_{2} )}}{{(\mu_{x}^{2} + \mu_{y}^{2} + c_{1} )(\delta_{x}^{2} + \delta_{y}^{2} + c_{2} )}} \\ \end{aligned}$$

where *M* is the MV CT image, and *K* is the kV CT image. And *G*(·) is the generated image of the given image in GAN, so that *G*(*M*) is the predicted synthetic kV CT image.

### Dataset and data processing

All research was performed in accordance with relevant guidelines and regulations. This study protocol was approved by the ethics committee/ institutional review board of the Yonsei University Severance Hospital, Korea (4-2022-0384), which waived the need for informed patient consent to the use of patient images. The patient cohort for training Networks 1 and 2 consisted of 90 brain and head-and-neck (HN) cancer cases who were treated by radiation therapy throughout two advanced TomoTherapy units (Radixact, Accuray Incorporated, USA). Of 90 scans, 72 scans had metal-contaminated kV CT slices. Network 1 trained the pairs of MV and kV CT image from 90 scans (80 and 10 scans for training and validating, respectively). Then, the trained Network 1 generated the synthetic kV CT images for 90 scans. Of 90 scans, Network 2 employed 72 scans containing metal-contaminated kV CT slices in network training. To validate the performance of Network 2 in charge of the actual MAR process, the additional 10 planning kV CT scans, including the MAR processed images provided by the Canon CT SEMAR algorithm, were applied to evaluating Network 2.

We picked out the patient cases treated by TomoTherapy units that obtained MV CT images for image-guided radiotherapy. The frequency of MV CT acquisition varies, depending on treatment type, which were obtained in the first fraction of treatment in most cases. The MV CT scan at the first fraction of the treatment was adopted for training Network 1 to maximize the image similarity between the planning kV CT and the MV CT images. The planning kV CT scans were acquired by Canon Aquilion LB CT simulator (Canon Medical Systems Coporation, Japan). The voxel spacing of the planning CT and TomoTherapy MV CT was 1.06 × 1.06 × 3 mm^3^, and 0.76 × 0.76 × 4 mm^3^, respectively, where both MV and kV CT images had the same 2D matrix, 512 × 512. The kV CT images were resampled to the MV CT images, such that the MV and kV CT images have the same size and resolution. The paired MV and kV CT images passed through the rigid and cubic B-spline-based deformable image registration (DIR) to further enhance the similarity. The DIR was performed by an open-source software Plastimatch (http://www.plastimatch.org) with a three-layered multi-resolution approach, where mutual information (MI) and mean-squared-error (MSE) were used as image similarity metrics in DIR for multi-modal and monomodal images, respectively.

**Figure 3:**
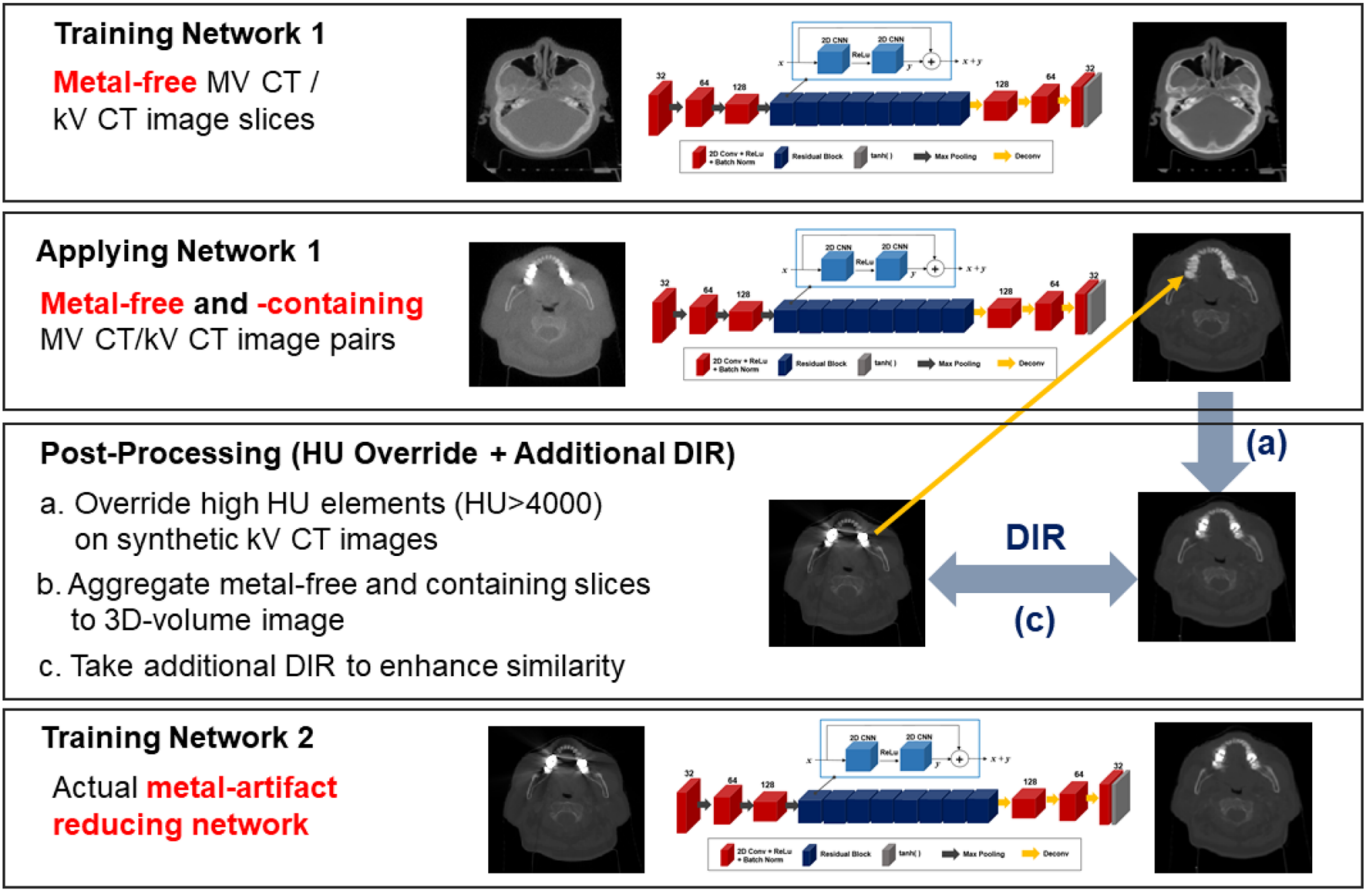
Flowchart of the additional image processing to the generated synthetic kV CT to improve image similarity between the prospective input and output images in Network 2.

As stated above, the actual MAR processing was performed in Network 2, while Network 1 was no longer engaged. Hence, to succeed in the MAR procedure, the metal-artifact-free images generated from Network 1 had to be as similar to the given kV CT images as possible, except for the metal contaminations. To achieve the goal, we conducted pre-processing (excluding kV CT image with high intensity (> 3000 HU)) and post-processing (intensity override and additional DIR) in training and inferring Network 1. Some kV CT images in Network 1 had metal artifacts unlike the paired MV CT images, which generated undesirable synthetic kV CT images. As a pre-processing, Network 1 trained the 2D slices with the MV-kV CT pair of images whose maximum kV CT intensity in the Hounsfield Unit (HU) is less than or equal to 3000 HU. Then, inference of Network 1 encompassed the removed 2D kV CT image slices (> 3000HU). The generated kV CT images did not describe the high intensity regions (> 3000 HU), which were overridden by those in the corresponding synthetic kV CT voxels as a post-processing. Furthermore, additional DIR operation was executed for the synthetic and true kV CT images. Unlike the initial DIR for multi-modal MV and kV CT images, the additional DIR for mono-modal images was expected to further enhance the image similarity in the synthetic and true kV CT images used for training Network 2. The pre- and post-processing operations were specified in Fig. 3.

### Implementation and evaluation

The two sequential networks with GAN were implemented in TensorFlow 1.14 (http://www.tensorflow.org) and Python 3.6 (http://www.python.org) on a personal workstation with an accelerated GPU (Nvidia GPX Titan X). The input and output images of the deep neural network were 2D axial images with 512 × 512 voxels. The intensity of the original CT images defined as an integer in the Hounsfield Unit was normalized such that the intensity of the input of the network ranged from -1 to 1 for training. The Adam optimizer was used for training the network with a mini-batch size of 3 by a learning rate of 2 × 10^–4^. The number of epochs was set to be 100 for both Network 1 and Network 2, which was empirically determined based upon the loss function values from validation and test dataset combined with the learning rates. To increase the number of images in training the network, we augmented the paired MV and kV CT images (used for training phase) by flipping (left–right), rotating (randomly from −10° to 10°), and translation (randomly $\pm$ 20 voxels in horizontal and vertical directions), which resulted in doubling and tripling the number of images in training Network 1 and Network 2, respectively.

The resulting synthetic kV CT images by Network 1, and additional pre- and post-processing were compared against true kV CT images for the metal-free slices only before training Network 2 in terms of mean-absolute-error (MAE), and SSIM. The image similarity metrics were quantified in 2D slice as the images were trained in the 2D-based CNN. Also, the image quality of the generated metal-artifact-reduced images from Network 2 was assessed in two aspects in this work: degree of metal artifact removal in a qualitative manner, and preservation of detailed imaging information post-correction (tissue and tissue-bone contrasts) in a quantitative manner. The second aspect, preservation of image details such as imaging contrast and soft-tissue information, was quantitatively analyzed in MAE and SSIM for 2D slices with high maximum HU (> 3000), while not encompassing severe metal artifacts. The extent of metal artifact suppression, which is the first aspect, was visually investigated for 2D slices, including explicit metal-contaminated artifacts, compared against the true kV CT, and the artifact-corrected image from commercial software, provided by Canon CT SEMAR algorithm.

## Results

### Image similarity between true kV CT and generated kV CT from Network 1

**Figure 4:**
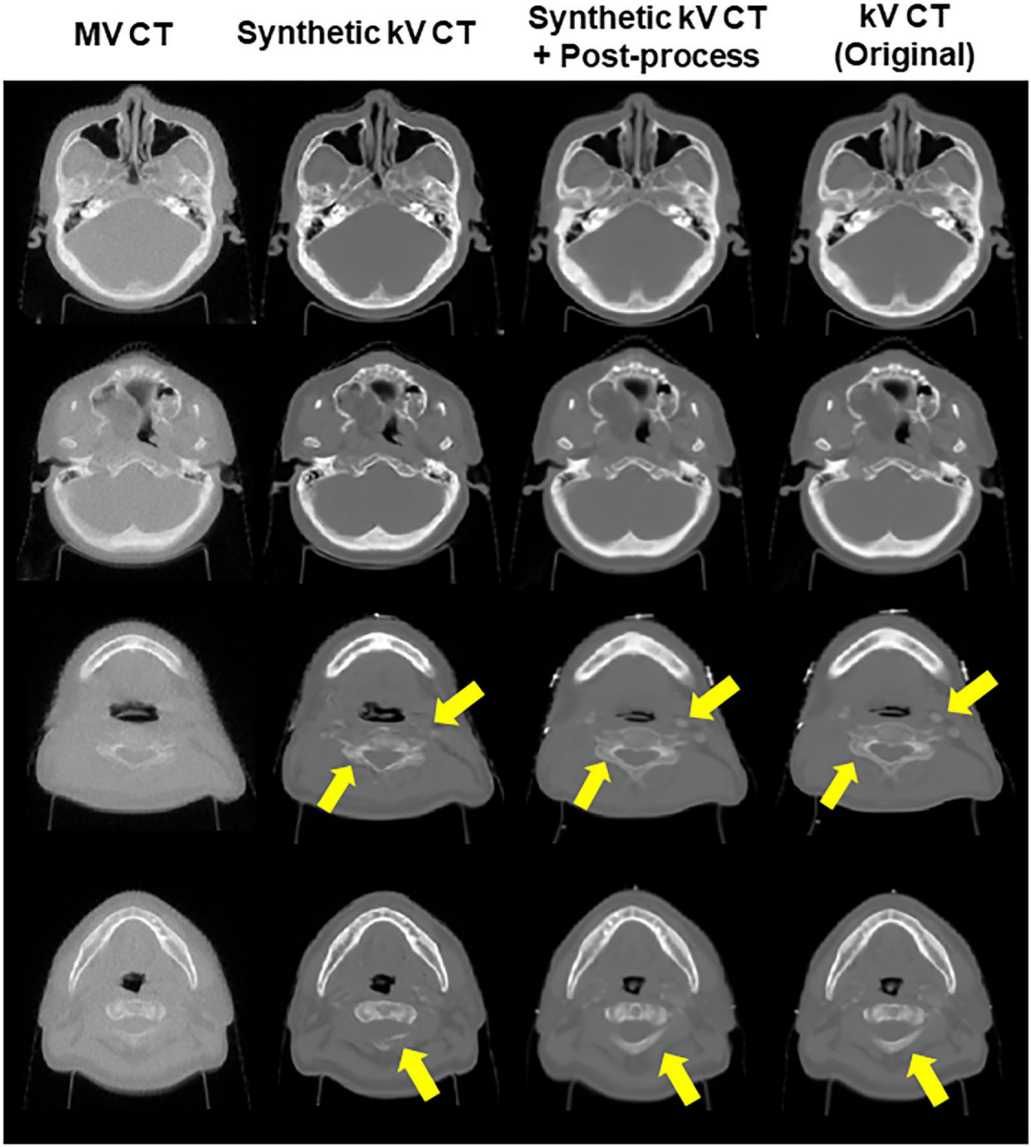
The results of Network1 (Synthetic kV CT) from MV CT and the images through additional processing (Synthetic kV CT + Additional Process) for 2D slices without containing metal elements, compared against true kV CT (MV CT [−1000 HU, 750 HU], kV CT: [−1000 HU, 1500 HU]).

**Figure 5:**
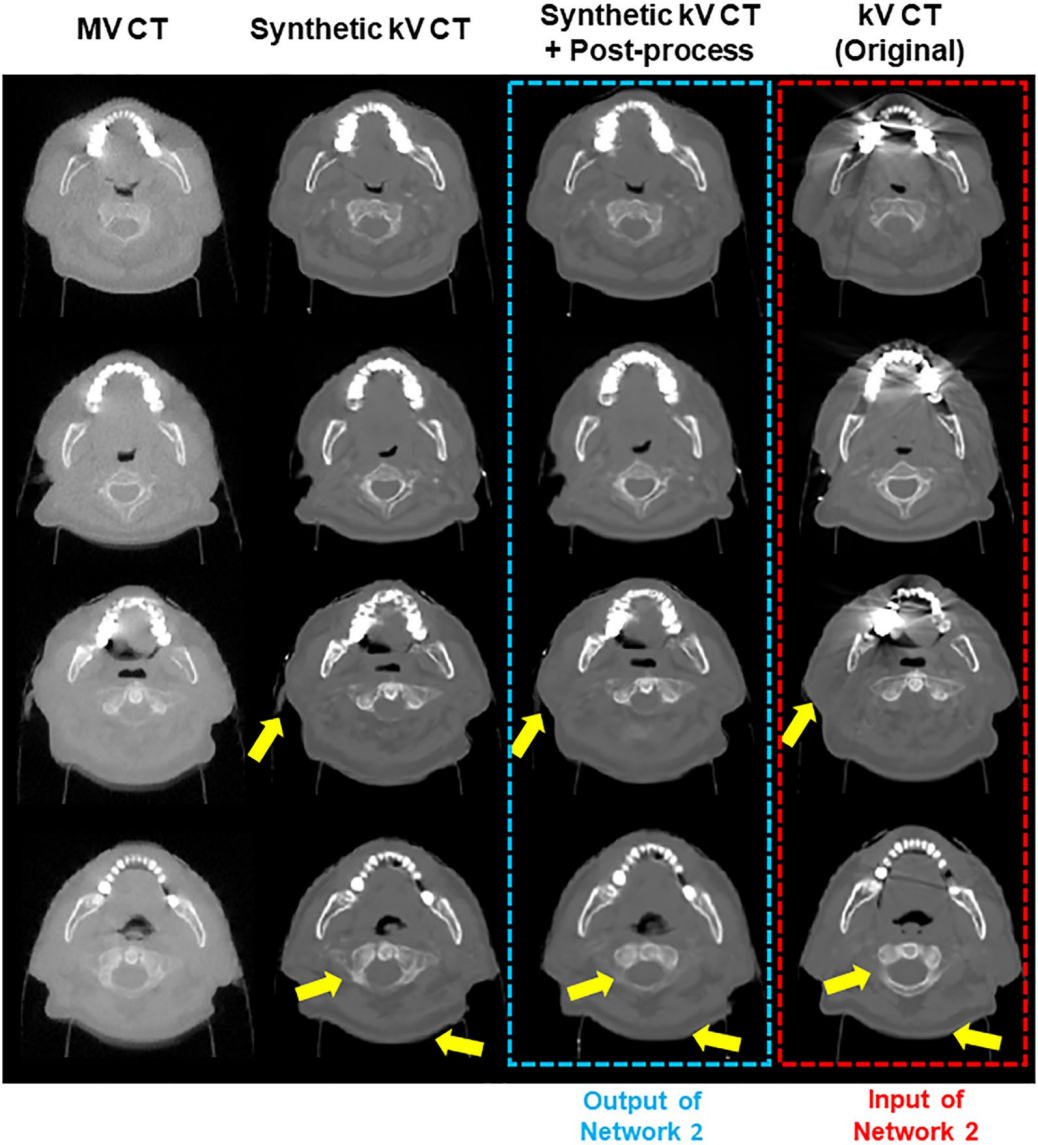
The results of Network1 (Synthetic kV CT) from MV CT and the images through additional processing (Synthetic kV CT + Additional Process) for 2D slices containing metal elements, compared against true metal-contaminated kV CT (MV CT [−1000 HU,750 HU], kV CT: [−1000 HU, 1500 HU]).

Figures 4 and 5 show the synthetic kV CT images generated from Network 1, and those after additional processing, besides true kV/MV CT images. The images in Fig. 4 were the slices without containing metal-associated elements. Network 1 was able to predict the qualified synthetic kV CT images, as seen in the first and second columns of Fig. 4. The synthetic kV CT images throughout Network 1 were sufficiently analogous to the true kV CT images, which did not easily discern. The third and fourth columns of Fig. 4 revealed that the generated images were not sufficiently similar to the true kV CT in imaging details, as indicated by the arrows in yellow. Table 1 provides the numerical results about image similarity between the generated and true kV CT images, including the degree of benefits from additional processing. The initial image similarity quantified by SSIM and MAE between different imaging modalities, MV and kV CT, were also given to see if the deep neural network could enhance the image similarity. The synthetic kV CT image from Network 1 got much closer to the true kV CT, yielding over SSIM of 0.967 and MAE of 56.6 HU. The additional processing turned out to be effective numerically as it promoted the similarity to SSIM of 0.997 and MAE of 10.2 HU.

**Table 1:** Numerical analysis about image similarity between kV CT and synthetic kV CT, and the effect from additional processing to image similarity.

	MV versus kV CT	Synthetic kV CT versus true kV CT	Synthetic kV CT + additional process versus true kV CT
SSIM	0.796	0.967	0.997
MAE	123.4HU	56.6 HU	10.2 HU

Figure 5 illustrates images, when applying the trained Network 1 to the 2D slices including metal-associated elements. As noted, MV CT is insensitive to metal artifacts though the corresponding kV CT slices were contaminated by the artifacts. In most cases, as shown in images in the first and second columns of Fig. 5, synthetic CT images produced from Network 1 were quite similar to true kV CT images in terms of surface shape regions that were not affected by metal-associated artifacts. Some of the synthetic kV images were not similar enough to the true kV CT images as seen in the third and fourth columns of Fig. 5. The images in the third row of Fig. 5 demonstrated that the additional DIR helped enhance the similarity as indicated by arrows in yellow in such cases. As the true kV CT images contained metal artifacts, the quantitative analysis of the artifact-free kV CT images was not performed. The kV CT images in the fourth (box in red) and third rows (box in blue) were then plugged into the input and output images for training Network 2.

### Performance of network 2 in charge of actual MAR

**Figure 6:**
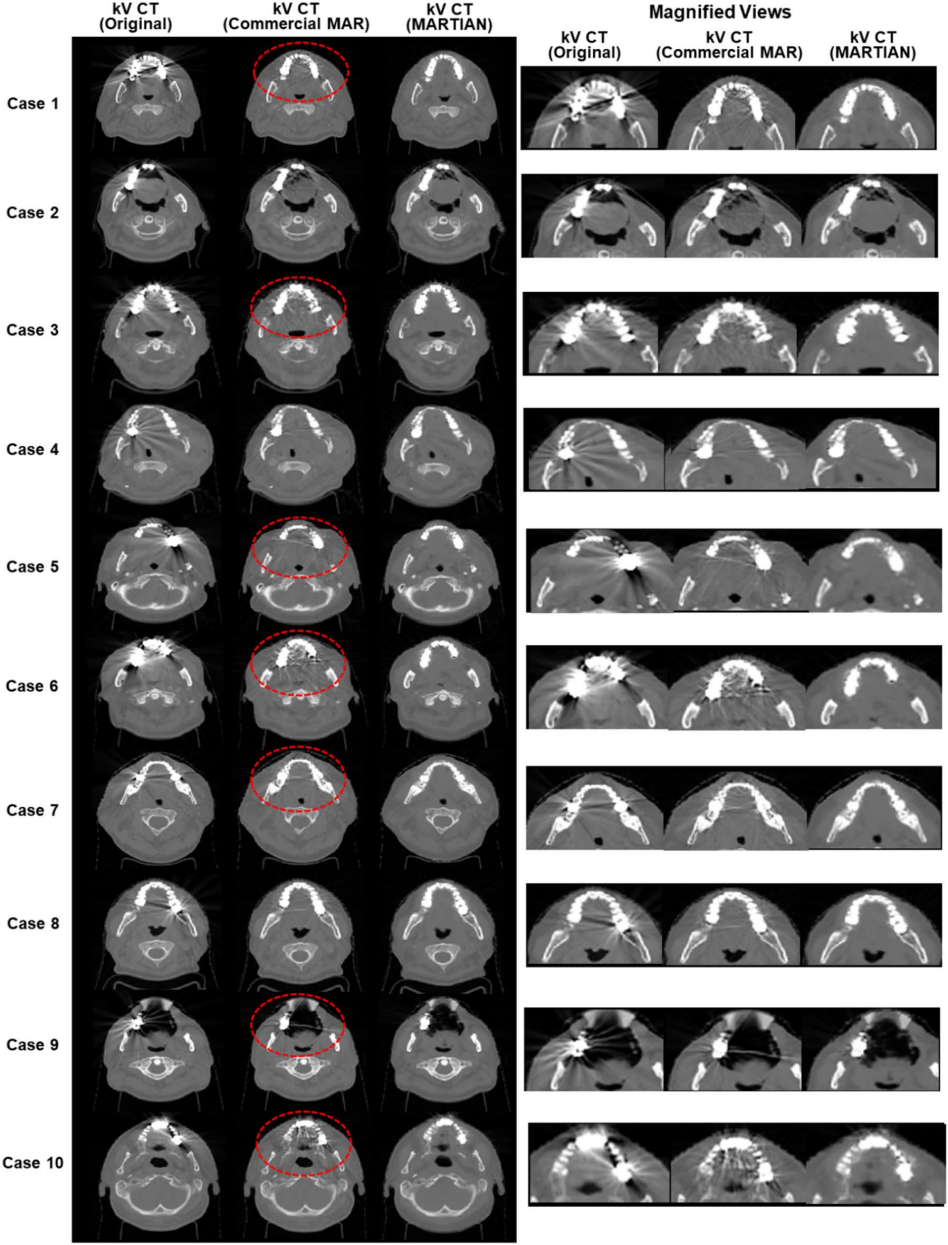
Resulting images from two-step sequential deep neural network for 10 test cases (third column), compared against metal contaminated true kV CT and kV CT images from commercial MAR (Canon SEMAR) (kV CT: [−1000 HU, 1500 HU]).

Network 2 trained the convolutional neural network with pairs of two kV CT images: metal-contaminated, original kV CT and artifact-free synthetic, post-processed kV CT images. Figure 6 visually investigated the performance of the proposed MAR framework (Network 2) for the ten testing cases by comparing the resulting kV CT images against the original kV CT and kV CT processed by the commercial MAR software. It can be seen that the commercial MAR software worked out partially, while the extent of artifact reduction depended on the clinical cases. The metal artifacts were successfully removed, while still leaving slight streak artifacts and dark bands. Occasionally, the remaining streak artifacts created scattered noise, as indicated by rounds in red in Fig. 6. The deep neural network based on the GAN architecture with CNN’s successfully controlled the metal artifacts in all testing cases, relative to the commercial MAR software, as seen in the third row of Fig. 6. The network parameters used in this work were well manipulated to preserve imaging details and tissue-bone contrasts, as seen in the 10 testing cases.

**Figure 7:**
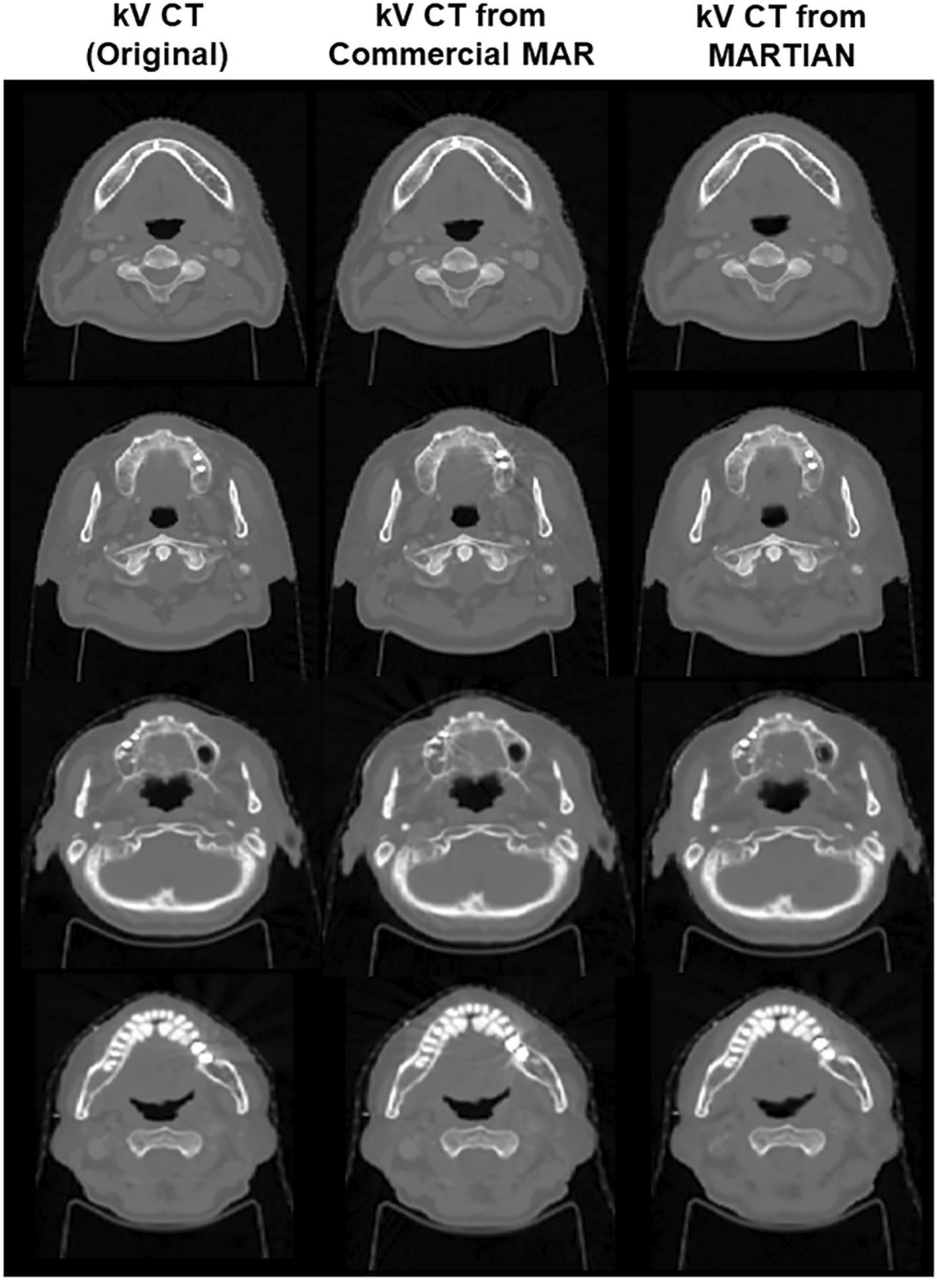
The predicted kV CT images from the proposed (third row) for 2D slices with high density (HU > 4000) and no containing metal-contaminated artifacts as seen in the true kV CT images (first row), and kV CT from the commercial MAR program (kV CT: [−1000 HU, 1500 HU]).

The difficulty of quantitative evaluation for the MAR framework lies in the fact that no ground-truth kV CT image perfectly removed the metal-associated artifacts. The visual inspection helped look into how the proposed framework could remove the metal artifacts. It did not, however, proceed to confirm how the MAR framework affected the soft tissue or bone regions adjacent to the metal-associated elements. Thus, the quantitative analysis to see the effect of the MAR framework on the soft tissue and bone regions adjacent to the metal-associated elements was performed in the selected 2D slices with high-density (HU > 3000), but not containing severe metal artifacts such as dark bands and streak artifacts, as seen in the first row of Fig. 7. Figure 7 showed that the kV CT images from CNN slightly smoothed the soft-tissue elements, compared against the true kV CT images. The extent of smoothness was not too excessive to undermine the contrast between soft tissue and bone, and the detailed information about the soft tissue. Table 2 provides the quantitative analysis of the imaging similarity between true kV CT and MAR-processed images from the commercial software. The resulting kV CT images from MARTIAN has SSIM of 0.995 and MAE of 20.2 HU, relative to the true kV CT images, which were quite competitive results in comparison with the commercial MAR software having SSIM of 0.997. The slight compromise in image similarity might have occurred since the trained network focused on removing the metal artifacts, followed by an inevitable influence in the resulting images at the predicted level.

**Table 2:** Numerical information about image similarity of resulting images throughout Network 2 for slices not containing severe metal artifacts, and having max. HU > 4000.

	kV CT (original) versus kV CT from commercial MAR	kV CT (original) versus kV CT from our proposed
SSIM	0.997	0.995
MAE	16.7 HU	20.2 HU

## Discussion

Deep learning with a convolutional neural network has become a powerful computational tool for medical image processing, such as segmentation, reconstruction, and translation. However, stretching it to artifact correction remains challenging as most of the network architectures in CNN are categorized as supervised learning that demands the artifact-free image as an output in training a network for artifact correction. The notion of multi-modal imaging with MV CT was adopted for reducing metal artifacts in the kV CT images, expecting the different physical characteristics of each medical imaging modality to be beneficial for overcoming the underlying shortcoming of supervised learning. By getting MV CT images robust to metal artifacts involved in deep neural network, we constructed a new two-step sequential network, named MARTIAN. The first deep neural network (Network 1) successfully generated synthetic metal artifact-free kV CT images from MV CT images, which were plugged into the output images for the corresponding metal-contaminated original kV CT images in training the subsequent second network (Network 2). Bringing MV CT image for MAR of kV CT image is not a new idea. Several previous studies tried to reduce metal artifacts of the kV CT using the MV CT images^42–44^. The direct MV CT to kV CT conversion discussed in such literatures relied on analytic approach that match the image intensity between MV and kV CT images. The resulting images could possess the disadvantages of the MV CT images, which are vulnerable to the scattering, followed by much less contrast than kV CT images. The learning-based method that generates the artifact-free kV CT image first from MV CT images may overcome these shortcomings. To our best knowledge, incorporating the MV CT into artificial intelligence (AI)-driven MAR has not been studied.

The framework of MARTIAN, where the second network in charge of actual artifact correction takes the synthetic images produced from the first network, was able to encounter challenges in the reliability of the artifact correction. Namely, the first network, Network 1, generating the synthetic kV CT from MV CT images may not be prepared for training Network 2 for the actual MAR. We specified the obstacles in two aspects. First, when training Network 1 in this work, the slices containing metal (high density) regions were excluded as those pairs of images did not offer any benefit in generating the artifact-free kV CT images. In contrast, the slices encompassing metal elements are required to conduct MAR.Intensity override easily solved this problem, in which the high-density region of generated synthetic kV CT images was overridden with the true-kV CT images when applying Network 1 to the metal-containing 2D slices. Second, before training Network 1, the DIR between MV and kV CT images was conducted. The multi-modal DIR does not generally guarantee registration accuracy, possibly leading to the sub-optimal image similarity in the generated synthetic images against the true kV CT. Thus, additional DIR process was performed with the synthetic, density-overridden kV CT and true kV CT images. The quantitative analysis of image similarity between the synthetic and true kV CT images demonstrated that the two extra processing was effective, which enlarged SSIM from 0.967 to 0.997 and lowered MAE from 56.6 HU to 10.2 HU.

Network 2 conducted training with the metal-contaminated true kV CT images as input, and synthetic, additionally processed kV CT images as output. Importantly, the images employed in training Network 2 were not overlapped with those trained for Network 1. The trained Network 2 was tested with the ten independent testing cases. As stated, the performance of the proposed MAR strategy was evaluated in 1) degree of reduction of metal artifacts for 2D slices with explicit metal artifacts, and 2) preservation of detailed imaging information for 2D slices without explicit metal artifacts. Throughout quantitative analysis for the second criterion, compared against the commercial MAR, the resulting images from MARTIAN had very competent results in image similarity to the true kV CT. The slight compromise in image quality was expected as the network was inherently enforced to remove the unwanted metal artifacts. The network essentially emphasizing the elimination of metal artifacts would have affected any other regions than the metal-associated elements, finally mitigating the image contrast or details slightly. Regarding the first criterion, in the extent of metal artifact suppression, our proposed strategy was superior to the commercial software, Canon SEMAR. The commercial software controlled the metal artifacts well in many testing cases. The degree of reduction, however, was inconsistent, varying from case to case. The remaining steaking artifacts caused troubles in image quality at times. It was important to note that the proposed framework performed consistently, and eliminated the metal artifacts in kV CT more effectively, while not damaging the detailed soft-tissue information adjacent to the metal elements.

The proposed work has a couple of limitations to be discussed. The verification of the proposed framework must be challenging as there were no ground-truth kV CT images due to the same reason that made the artifact correction challenging throughout supervised learning. Thus, the resulting artifact-free kV CT images were visually investigated against true kV CT images and MAR-processed images from the commercial software. The quantitative analysis was executed for the metal-artifact-free slices only. Second, all the frameworks developed in this work paid attention to the application of the brain or head-and-neck kV CT scans. Generally, the deep convolutional neural network is likely to conform to what types of data were used in training. Thus, site-specific training scheme would be required to extend this framework to the other body sites. Third, the performance of the deep neural network highly depends on the magnitude of data. This work employed 90 and 72 cases for training Network 1 and Network 2. The number of 2D slices available for Network 2 got more reduced. Though the number of samples was partially compensated by augmentation, it might have constrained the performance. To be able to enhance the accuracy and be clinically useful, the training data would have to be bigger.

Comparing the proposed work to the previous deep learning-based MAR approach, which generates artificial artifact-contaminated images, would be interesting. It is believed that our proposed work would be the more compelling strategy for MAR in several aspects. The previous learning-based approach might not be able to describe non-linear and unpredicted features of the artifact distributions on the kV CT images. This strategy was possible by manipulating the sinogram of the artifact-free image, while it may not be achievable for the other types of medical imaging modalities. It implies that the approach is limited in expandability besides reality. Contrarily, our proposed model focused on generating artifact-free synthetic images from actual clinical data acquired by a different modality, while leaving the original kV CT images intact. Additional post-processing was conducted to further reduce the possible propagation of discrepancy between multi-modal images, yielding a high degree of image similarity. Given multi-modal images that are mutually beneficial in physical characteristics available, the two-step framework might be more powerful with respect to artifact correction of the medical images.

## Conclusion

MARTIAN was characterized as a combination of a deep convolutional neural network and the notion of multi-modal imaging for the purpose of metal artifact suppression in kV CT. MV CT images known to be robust against metal artifacts unlike kV CT were involved in the framework to produce artifact-free kV CT images, which were brought into the subsequent deep neural network for actual MAR. To make artifact correction more appropriate, the synthetic kV CT images throughout the first network were additionally processed, leading to enhancing image similarity to the true kV CT images. Training the second network with such pairs of metal contaminated and artifact-free kV CT images successfully suppressed metal artifacts in kV CT, which outperformed the commercial MAR software without compromising the image contrast and detailed information.

## Data Availability

The datasets generated during the current study will be available from the corresponding author on reasonable request.
